# Integrative phenotypic and gene expression data identify myostatin as a muscle growth inhibitor in Chinese shrimp *Fenneropenaeus chinensis*

**DOI:** 10.1038/s41598-020-61382-8

**Published:** 2020-04-06

**Authors:** Jie Kong, Yunjun Yan, Xia Lu, Sheng Luan, Xianhong Meng, Ping Dai, Baolong Chen, Baoxiang Cao, Guangfeng Qiang, Kun Luo

**Affiliations:** 1 Wuxi Fisheries College, Nanjing Agricultural University, Wuxi, 214081 PR China; 2 Key Laboratory of Sustainable Utilization of Marine Fisheries Resources, Ministry of Agriculture, Yellow Sea Fisheries Research Institute, Chinese Academy of Fishery Sciences, Qingdao, 266071 China; 3 Laboratory for Marine Fisheries Science and Food Production Processes, Qingdao National Laboratory for Marine Science and Technology, Qingdao, 266071 China

**Keywords:** Animal breeding, Gene expression, Heritable quantitative trait, Quantitative trait

## Abstract

Growth traits, largely determined by muscle growth, are the most critical economic traits in shrimp breeding. Myostatin (*Mstn*) is a conserved inhibitor of muscle growth in vertebrates, but until now solid evidence supporting a similar function of *Mstn* in invertebrates has been lacking. In the present study, we examined the *Mstn* expression along with growth trait data in a *Fenneropenaeus chinensis* population, to establish a potential correlation between *Mstn* and growth. The heritabilities of *FcMstn* expression, body weight at 190 days of culture, body weight and length at 230 days of culture, and average daily gain were estimated using 773 individuals and a thirteen-generation pedigree. The results showed *FcMstn* expression was negatively correlated with the growth traits, and the mean *FcMstn* expression in females was significantly lower than that of males, indicating *Mstn* negatively regulates muscle growth in shrimp, and its lower expression may underscore the faster growth of females. Low heritabilities were detected for *FcMstn* expression, suggesting that the expression of *Mstn* might be heritable in shrimp. These results provide strong support for a growth inhibitory function of *Mstn* in *F. chinensis*, and suggest a potential method for selective breeding of this species without substantial experimental resources and labor force.

## Introduction

Shrimp is an important aquaculture species with high single output values, which takes a great role in the economic development of the world. To improve the production has always been the important goal in shrimp farming. Faster growth is essential for increasing production, which could make for more economic benefits and sustainable development for shrimp breeding. Selective breeding is an important propelling force for the development of efficient and sustainable production^1^. In the beginning, mass selection was the most commonly used method for selection breeding in aquaculture, because it was easy to manipulate^2^. Afterwards, family-based selection became the alternative methods in aquatic species due to its effect for all types of traits^3^. The family-based selective breeding programs have improved the target traits a lot and made great contributions to the development of shrimp industry^4–6^. However, there were still some difficulties in family-based selective breeding, such as the high investment and operational costs^7^, and long and complex test periods. Therefore, new methods and technologies are urgently required for accelerating the genetic improvement of important traits in shrimp.

Along with the development of molecular biology technology, marker assisted selection (MAS) and genome-wide selection (GWS) were suggested as effective methods for selecting for target traits^8,9^. However, the markers used for MAS need to be closely linkaged with the larger effective quantitative trait locus (QTL) of the target trait, and GWS needs large number of markers to be genotyped in large scale samples. Although the costs of MAS and GWS technologies are gradually decreasing as new breakthroughs in methodology, they are still unacceptable and unsustainable for shrimp selection programs. Consequently, we need a quick and cheap method for selective breeding.

Robinson *et al*.^10^ suggested to use the gene expression profiles with larger sample sizes as an indirect parameter for the target traits as an alternative method for selective breeding. Oleksiak *et al*.^11^ observed statistically significant differences in gene expression within and among populations in fish, and Robinson and Hayes^12^ have modeled the use of gene expression as an alternative method for improving disease resistance in selective breeding of fish. Gene expression has been studied as heritable and quantitative traits in genetic studies, and quantitative geneticists have long been interested in the heritability of the gene expression^13^. To dissect such traits, a fundamental question is what proportion of the variation of the gene expression among individuals in a population can be attributed to genetic factors, but rather than environmental and random differences^14^. In the previous studies, a significant heritable component of gene expression has been found in yeast^15^, mice^16^, flies^17^, fish^18^ and human^19^. However, because of experimental shortfalls most of these studies did not provide a direct estimate of heritability, mainly due to the small sample scales and lower genetic tie among individuals.

Chinese shrimp, *Fenneropenaeus chinensis*, is one of the most valuable maricultural species. Our group has initiated a selective breeding program for *F. chinensis* in 2004 using the family-based selection method to increase production^6^. Until now, the selection for *F. chinensis* is still in progress and the population has been selected for thirteen generations. The deep pedigree enables a high level of genetic tie among the families and individuals. Consequently, this shrimp population provided ideal materials for heritability estimate of gene expression.

Muscle tissue as the main edible part accounted for the majority mass of shrimp tissue, which plays a decisive role in shrimp growth. Myostatin (*Mstn*) is predominantly expressed in the muscle tissues and essentially inhibits muscle growth in vertebrates. For example, there was a dramatic increase in the skeletal muscle mass after knocking out *Mstn* in mouse^20^, and the muscle size of the cattle with function loss of *Mstn* would increase 20% compared to the normal cows^21^. In addition, “double-muscled” phenotype was observed in sheep and cattle with mutation in *Mstn*^22–25^. Because *Mstn* affected muscle mass and the increased musculature would transform into higher production, it also attracted much research interest on its function in invertebrates, among which including shrimp, such as *Penaeus monodon*^26^, *Litopenaeus vannamei*^27^, *F. merguiensis*^27–29^ and *F. chinensis* (the data has not been published). Preliminary results suggest that *Mstn* also involved in muscle growth in shrimp. However, until now solid evidence supporting a similar function of *Mstn* has been lacking in invertebrates.

The main objectives of the present study were to establish a potential correlation between *Mstn* and muscle growth with large scale sample in shrimp. We are also very curious to know whether the expression of *Mstn* was heritable, how heritable is *Mstn*, and how is variation of *Mstn* expression within the population of shrimp. Consequently, we examined the expression of *Mstn* along with growth trait data in 773 individuals from 44 full-sib families of the population of *F. chinensis*. In order to investigate the potential use of the expression of *Mstn* as an indirect parameter for growth traits as an alternative method for selective breeding, the expression level of *Mstn* was treated as a quantitative trait and its heritability was estimated using a thirteen-generation deep pedigree. In addition, we estimated the heritabilities of body weight (BW1) at 190 days of culture (DOC), body weight (BW2) and body length (BL2) at 230 DOC, and average daily gain (ADG) for this population. The results would provide important information for use of *Mstn* in the breeding of shrimp.

## Results

### Descriptive statistics

**Figure 1:**
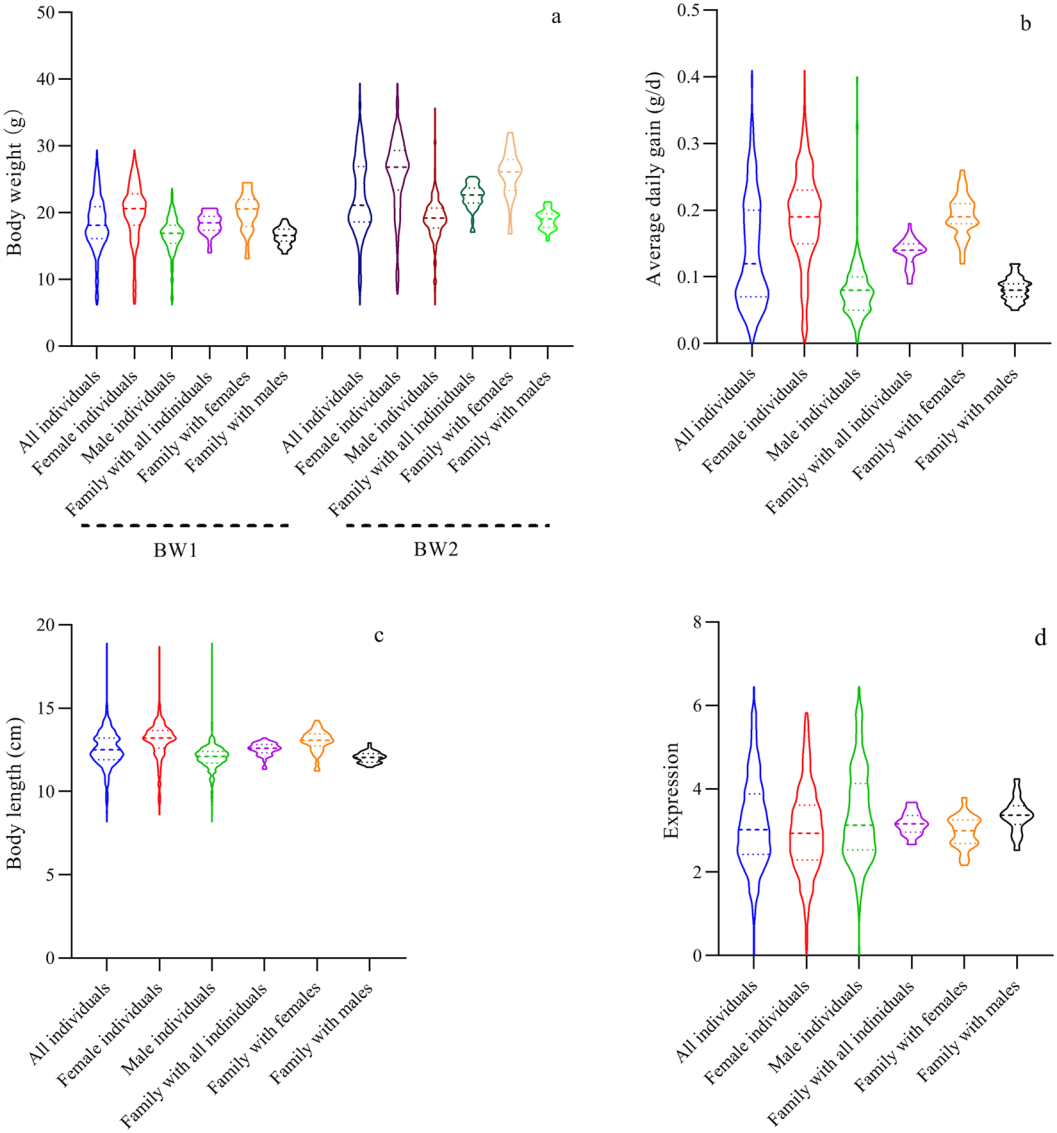
Violin plots of the body weight (BW1 and BW2) (**a**), average daily gain (ADG) (**b**), body length (BL2) (**c**), and the expression of *FcMstn* (**d**) at individual and family level.

Finally, a total of 773 individual records from 44 full-sib families, including 383 females and 390 males were analyzed in the present study. The number of observations, mean value, maximum and minimum, standard deviation and coefficient of variation for BW1, BW2 and BL2, ADG, and *FcMstn* expression at individual (all individuals, females, and males, respectively) and family (family mean with all individuals, females, and males, respectively) levels are shown in Table 1. The 25th percentiles, median percentiles, 75th percentiles, minima and maxima of the five traits at individual and family levels are displayed in Fig. 1. The results revealed the BW1, BW2, ADG, and *FcMstn* expression values both varied substantially among the individuals and families, but the BL2 has lower variance at the two levels. However, the variance was higher when analyzed at the individual level than the family level as indicated by the higher standard deviation (SD) and coefficient of variation (CV) for all the traits (Table 1). For the growth traits (BW1, BW2, BL2, and ADG), the means of females were significantly higher than those of males at both of individual and family levels (*P* < 0.001). However, for the *FcMstn* expression, the mean of females was significantly lower than that of males at the two levels (*P* < 0.001).

**Table 1:** Numbers of samples/families (N) and the means, minima, maxima, standard deviations, and coefficients variation of the five traits.

Traits		N	Minimum	Maximum	Mean	Standard deviation	Coefficient variation (%)
Body Weight 1 (BW1, g)	All individuals	773	6.20	29.40	18.33	4.03	22.00
	Female individuals	383	6.30	29.40	20.09	4.33	21.55
	Male individuals	390	6.20	23.70	16.60	2.77	16.69
	All families	44	13.97	20.68	18.32	1.58	8.62
	Female families	44	13.10	24.52	20.11	2.78	13.82
	Male families	44	13.81	19.11	16.49	1.29	7.82
Body Weight 2 (BW2, g)	All individuals	773	6.20	39.40	22.43	5.68	25.32
	Female individuals	383	7.80	39.40	25.83	5.63	21.80
	Male individuals	390	6.20	35.70	19.09	3.23	16.92
	All families	44	17.07	25.44	22.41	1.89	8.43
	Female families	44	16.83	32.03	25.84	3.43	13.27
	Male families	44	15.76	21.63	18.96	1.40	7.38
Average daily gain (ADG, g)	All individuals	773	0.00	0.41	0.14	0.08	57.14
	Female individuals	383	0.00	0.41	0.19	0.07	36.84
	Male individuals	390	0.00	0.40	0.08	0.05	60.55
	All families	44	0.09	0.18	0.14	0.02	14.29
	Female families	44	0.12	0.26	0.19	0.03	15.79
	Male families	44	0.05	0.12	0.08	0.02	25.00
Body length2 (BL2, cm)	All individuals	773	8.20	18.90	12.54	1.11	8.85
	Female individuals	383	8.60	18.70	13.03	1.13	8.67
	Male individuals	390	8.20	18.90	12.06	0.85	7.05
	All families	44	11.34	13.22	12.54	0.39	3.11
	Female families	44	11.24	14.28	13.03	0.66	5.07
	Male families	44	11.46	12.92	12.05	0.32	2.66
*FcMstn* expression	All individuals	773	0.00	6.46	3.17	1.09	34.50
	Female individuals	383	0.00	5.83	2.99	1.04	34.76
	Male individuals	390	0.00	6.46	3.35	1.12	33.37
	All families	44	2.67	3.69	3.17	0.26	8.30
	Female families	44	2.17	3.80	2.97	0.38	12.82
	Male families	44	2.53	4.24	3.36	0.40	11.78

### Variance components and heritability estimations

Estimates of the variance component and heritability for BW1, BW2, ADG, BL2, and *FcMstn* expression at all-individual, female-individual, and male-individual levels are provided in Table 2. For BW1, BW2, and BL2, the females have the highest heritabilities, followed by all individuals, but the males have the lowest heritabilities. Excepting for BL2 in males, the heritability estimates of the five traits (BW1, BW2, BL2, ADG, and *FcMstn* expression) were all significantly different from zero at the three levels (*P* < 0.05). According to the classification reported by Cardellino and Rovira^30^ (i.e., low, 0.05–0.19; medium, 0.20–0.44; high, 0.45–0.64; and very high, >0.65), the *FcMstn* expression have low heritabilities (0.05 ± 0.03, 0.06 ± 0.06, and 0.08 ± 0.06, respectively) at all-individual, female-individual, and male-individual levels; the heritability estimates of BW1 (0.75 ± 0.14) and BW2 (0.66 ± 0.13) in females were very high; the heritability of BL2 (0.59 ± 0.13) in females was high; the heritability estimates of BW1 (0.35 ± 0.09), BW2 (0.34 ± 0.09), BL2 (0.22 ± 0.07), and ADG (0.22 ± 0.08) in all individuals, and BW1 (0.25 ± 0.09), BW2 (0.26 ± 0.10), and ADG (0.44 ± 0.11) in males were medium. The heritabilities of BW1 were not significantly different from those of BW2 at all the three levels (*P* > 0.05). The heritability estimates of BW1, BW2, and BL2 in females were significantly greater than those in all individuals and males (*P* < 0.05), but there was no significant difference between all individuals and males (*P* > 0.05). For the heritabilities of ADG and *FcMstn* expression, there was no significant difference among the three levels (*P* > 0.05). For the five traits, the females were positively correlated with males (*P* < 0.05).

**Table 2:** Variance components and heritabilities of for the traits.

Traits		Variance components		Heritability		Genetic correlation
		${\sigma }_{a}^{2}$	${\sigma }_{e}^{2}$	${\sigma }_{p}^{2}$	${h}^{2}$ ± SE	r_g_
BW1	All individuals	4.86	8.86	13.72	0.35 ± 0.09^*,b^	
	Female individuals	15.88	5.23	21.11	0.75 ± 0.14^*,a^	0.39 ± 0.02^*^
	Male individuals	1.88	5.79	7.67	0.25 ± 0.09^*,b^	
BW2	All individuals	7.50	14.54	22.04	0.34 ± 0.09^*,b^	
	Female individuals	22.84	11.94	34.81	0.66 ± 0.13^*,a^	0.44 ± 0.02^*^
	Male individuals	2.81	7.89	10.71	0.26 ± 0.10^*,b^	
BL2	All individuals	0.23	0.80	1.02	0.22 ± 0.07^*,b^	
	Female individuals	0.81	0.57	1.38	0.59 ± 0.13^*,a^	0.36 ± 0.05^*^
	Male individuals	0.06	0.67	0.73	0.08 ± 0.07^b^	
ADG	All individuals	0.82E-03	0.29E-02	0.37E-02	0.22 ± 0.08^*^	
	Female individuals	0.90E-03	0.39E-02	0.48E-02	0.19 ± 0.09^*^	0.71 ± 0.04^*^
	Male individuals	0.12E-02	0.15E-02	0.27E-02	0.44 ± 0.11^*^	
	All individuals	82.92	1646.52	1729.40	0.05 ± 0.03	
*FcMstn* expression	Female individuals	79.68	1331.06	1410.70	0.06 ± 0.06	0.35 ± 0.05^*^
	Male individuals	157.74	1905.51	2063.20	0.08 ± 0.06	

*Estimate is significantly different from zero (*P* < 0.05). ^a,b^Represents the significant differences among the all-individual, female-individual, and male-individual levels of the same trait. The variance component terms are as follows: ${\sigma }_{a}^{2}$ is the additive genetic variance, ${\sigma }_{e}^{2}$ is the random residual error variance, and ${\sigma }_{p}^{2}$ is the phenotypic variance. r_g_, genetic correlation between female and male.

### Genetic and phenotypic correlation analysis

The correlation analysis based on phenotypic and breeding values between traits at all-individual, female-individual, and male-individual levels are given in Table 3. Importantly, there were significant negative genetic correlations between *FcMstn* expression and other growth traits (BW1, BW2, BL2, and ADG) at the three levels, excepting for the non-significant negative genetic correlation with ADG in males. Especially, there were extremely significant genetic correlation between *FcMstn* expression and BW1 (−0.91), BW2 (−0.97), BL2 (−0.98), and ADG (−0.84) in females, and there were also significant genetic correlation between *FcMstn* expression and BW1 (−0.86), BW2 (−0.85), and BL2 (−0.64) in males. In generally, there are significantly positive correlations between the growth traits, and r_g_ values were higher than r_p_ values; females have the highest r_g_ and r_p_ values, followed in all individuals, but the males have the lowest correlation values, and almost all of the correlations were significantly different from zero (Table 3). BW2 is significantly correlated with BL2 (r_g_ was 0.99, 1.00, and 0.91, respectively), BW1 (r_g_ was 0.97, 1.00, and 0.81, respectively) and ADG (r_g_ was 0.71, 0.94, and 0.63, respectively) in all individuals, females, and males. In addition, BW1 is significantly correlated with BL2 (r_g_ was 0.98, 1.00, and 0.84, respectively) in all individuals, females, and males, and BL2 is also significantly correlated with ADG (r_g_ was 0.76, 0.95, and 0.57, respectively) at the three levels.

**Table 3:** Genetic and phenotypic correlation between the five traits for all, female, and male individuals.

Traits		BW1	BW2	ADG	BL2	*FcMstn* expression
All individuals	**BW1**	–	0.94 ± 0.01^*^	0.38 ± 0.04^*^	0.83 ± 0.01^*^	**−0.30** ± 0.03^*^
	**BW2**	0.97 ± 0.02^*^	–	0.68 ± 0.02^*^	0.86 ± 0.01^*^	**−0.32** ± 0.03^*^
	**ADG**	0.52 ± 0.19^*^	0.71 ± 0.12^*^	–	0.55 ± 0.03^*^	**−0.21** ± 0.04^*^
	**BL2**	0.98 ± 0.02^*^	0.99 ± 0.01^*^	0.76 ± 0.12^*^	–	**−0.32** ± 0.03^*^
	***FcMstn*** **expression**	**−0.32** ± 0.09^*^	**−0.38** ± 0.09^*^	**−0.37** ± 0.04^*^	**−0.29** ± 0.03^*^	–
		**BW1**	**BW2**	**ADG**	**BL2**	***FcMstn*** **expression**
Female individuals	**BW1**	–	0.95 ± 0.01^*^	0.51 ± 0.04^*^	0.87 ± 0.01^*^	**−0.51** ± 0.04^*^
	**BW2**	1.00 ± 0.01^*^	–	0.75 ± 0.03^*^	0.92 ± 0.05^*^	**−0.54** ± 0.04^*^
	**ADG**	0.91 ± 0.11^*^	0.94 ± 0.07^*^	–	0.67 ± 0.03^*^	**−0.39** ± 0.04^*^
	**BL2**	1.00 ± 0.02^*^	1.00 ± 0.03^*^	0.95 ± 0.08^*^	–	**−0.52** ± 0.04^*^
	***FcMstn*** **expression**	**−0.91** ± 0.11^*^	**−0.97** ± 0.19^*^	**−0.84** ± 0.13^*^	**−0.98** ± 0.19^*^	–
		**BW1**	**BW2**	**ADG**	**BL2**	***FcMstn*** **expression**
Male individuals	**BW1**	–	0.89 ± 0.01^*^	0.12 ± 0.06^*^	0.71 ± 0.03^*^	**−0.20** ± 0.05^*^
	**BW2**	0.81 ± 0.09^*^	–	0.55 ± 0.04^*^	0.74 ± 0.02^*^	**−0.22** ± 0.05^*^
	**ADG**	0.05 ± 0.07	0.63 ± 0.16^*^	–	0.32 ± 0.05^*^	−0.10 ± 0.05
	**BL2**	0.84 ± 0.19^*^	0.91 ± 0.12^*^	0.57 ± 0.16^*^	–	−0.22 ± 0.05
	***FcMstn*** **expression**	**−0.86** ± 0.17^*^	**−0.85** ± 0.15^*^	−0.02 ± 0.05	**−0.64** ± 0.02*	–

*Estimate is highly significantly different from zero (*P* < 0.05). The phenotypic correlations are above the diagonal, and the genetic correlations are under the diagonal.

## Discussion

Growth traits, largely determined by muscle growth, are the most concerned economic traits in the shrimp breeding^31^. Sexual dimorphism for growth of shrimp is very distinct; specifically, females tend to grow faster and bigger than males, such as in *F. chinens*^6^, *L. vannamei*^4,32^, and *P. monodon*^33,34^. *Mstn* is a conserved inhibitor of muscle growth in vertebrates, but until now solid evidence supporting a similar function of *Mstn* in invertebrates has been lacking. In the present study, we firstly to establish a potential correlation between the expression of *Mstn* and growth using a large sample size in shrimp, containing the difference of its expression level between females and males. In addition, we firstly estimated the heritability of the *FcMstn* expression with the large sample size and a deep pedigree.

According to the classification reported by Cardellino and Rovira^30^, the growth trait has medium to high heritability (0.24–0.52) in shrimp^5,6,35,36^, which was consistent with our results, illustrating that the genetic factor played important role during their growing process. Some scholars pointed out that variation in gene expression may play more important roles than differences between variant forms of proteins for evolution^37^. The DNA region that affects gene expression is highly variable, containing more than 0.6% polymorphism^38^. The naturally occurring polymorphisms of nucleotides could affect the gene expression *in vivo*^39–41^. In the present study, the growth rates varied substantially among the individuals and among the families even their growth were selected for thirteen generations, and females generally grew faster than males (Table 1). This phenomenon may be related to different expression levels of some genes among the individuals and between females and males.

In the previous studies of *F. merguiensis*^29^ and *F. chinens* (the results would be published in our other study named “Identification of myostatin and investigation of its inhibitory function on myogenesis and muscle growth in Chinese Shrimp, *Fenneropenaeus chinensis*”), significantly higher expression level of *Mstn* in muscle tissue was observed in the smaller shrimp than in the larger shrimp with about 10 individuals for each group. In the present study, we analyzed the expression of *FcMstn* in a large-scale sample (383 females and 390 males), and the results showed its expression varied substantially among individuals and among families (Table 1), which has provided important precondition and foundation for growth selection by analyzing its expression. Importantly, significantly negative genetic correlation was detected between the expression level of *FcMstn* and the growth traits (BW1, BW2, BL2, and ADG), especially in the females (r_g_ ranged from −0.84 to −0.98) (Table 3). In addition, the mean expression of *Mstn* in females was significantly lower than that of males at individual and family levels (*P* < 0.001). These results further demonstrate that *Mstn* negatively regulates muscle growth in shrimp, and its lower expression may underscore the faster growth of females. On this point, the results were found out some resemblance to the study on *F. merguiensis Mstn* that knock-down of *FmMstn* gene by RNAi can cause a stronger muscle development in *F. merguiensis*^29^. However, the study on *P. monodon Mstn* showed that shrimp with reduced expression level of *Mstn* mRNA displayed a slow growth rate compared with the control groups^26^, which might be caused by the effects of molt from the small sample scale and short experiment period. Because silencing of *Mstn* increased premolt duration, but the higher *PmMstn* expression in premolt stage led to slow growth^26^. Zhuo *et al*.^29^ detected high polymorphism and identified several potential SNPs in *Mstn* of *F. merguiensis*. In addition, two alternative splicing isoforms of *Mstn* was found in *F. merguiensis*^29^ and *F. chinens* (the results would be published in our other study named “Identification of myostatin and investigation of its inhibitory function on myogenesis and muscle growth in Chinese Shrimp, *Fenneropenaeus chinensis*”). Shin *et al*.^42^ reported alternative splicing isoforms of *Mstn* could negatively regulate pro-myostatin processing in muscle cells and prevent *Mstn* mediated inhibition of myogenesis in avian species. Consequently, we deduced that the variation in the expression of *Mstn* affected the growth rate of the shrimp, which might result from the difference of polymorphic nucleotides and alternative splicing isoforms among the individuals/families and between females and males. In the future study, we will carry out research to further verify the relationship between the polymorphic nucleotides or alternative splicing isoforms and the expression level of *Mstn*.

To investigate the potential use of the *Mstn* expression as quantitative trait for improving growth by selective breeding in shrimp, the heritability of its expression was estimated in the present study. In the previous heritability studies of gene expression, they all suggested a strong component of differential expression among genotypes^15–19^. However, because of the small sample sizes and lower genetic tie among individuals, most of these studies did not provide a direct estimate of heritability. The previous studies demonstrated that sample sizes of 100 individuals were too small to support robust estimates of heritability^13,43^. In the present study, we used a large scale sample (773 individuals from 44 full-sib families) and a thirteen generation pedigree for estimating the heritability of the expression of *Mstn*. Low heritability estimations were detected for the expression of *Mstn* at all-individual, female-individual, and male-individual levels, suggesting that the expression of *Mstn* might be heritable in shrimp. In addition, the significantly negative correlated between the expression of *Mstn* and the body weight and average daily gain indicated that there was selective potential for improving growth performance by analyzing the expression of *Mstn* at early stage before substantial experimental resources and labor force are invested. In future study, we will use more stages including the juvenile at rapid growth stage to verify the heritability of the expression of *Mstn* and its correlation with later stages.

Traditionally, the heritability estimates for growth were mainly focused on harvest body weight in shrimp^6,35,44^. In the present study, the heritabilities of harvest body weight (BW1), body weight (BW2) and body length (BL2) of later stage, and average daily gain (ADG) were estimated for the G_13_ of *F. chinensis*. In our previous study, the heritabilities of harvest body weight for the G_3_-G_5_ of this population were 0.23–0.36, 0.22–0.36, 0.37–0.38 at all-individual, female-individual, and male-individual levels, respectively^6^, and the heritabilities in males were higher than those of females. In the present study, the heritabilities of BW1 for the G_13_ were 0.35, 0.75, and 0.25 at the three levels. There was no significant difference between the heritabilities of G_3_-G_5_ and that of G_13_ both at all-individual and male-individual levels, but the heritability of BW1 for the G_13_ was significantly higher than those of the G_3_-G_5_ in females^6^ (Table 2). In the present study, the heritabilities of BW1, BW2, and BL2 in females were also significantly higher than those in males, which were consistent with the results of previous studies in *M. rosenbergii*^45,46^. They also reported heritabilities were higher in females than those in males^45,47,48^. When the females and males were treated as a separate trait, they have significant positive genetic correlations (Table 2), indicating that the growth of females and males are controlled by the same genes, such as *Mstn*. There was no significant difference for heritability estimates between BW1 and BW2 at the three levels, and BW1 has significant genetic correlations with BW2, BL2, and ADG, indicating the later growth could be selected by the harvest body weight.

In summary, we analyzed the expression of *Mstn* along with growth trait data in 773 individuals (383 females and 390 males) from 44 full-sib families of a *F. chinensis* population, to establish a potential correlation between *Mstn* and growth in this study. In addition, the expression of *Mstn* was treated as a quantitative trait, and its heritability was estimated using these 773 samples and a thirteen generation pedigree. The results provide strong support for a growth inhibitory function of *Mstn* in shrimp, and its lower expression may underscore the faster growth of females. The result also demonstrated that the expression of *Mstn* might be heritable in shrimp, suggesting that there might be potential for improving growth performance by analyzing the expression of *Mstn* at early stage. The heritabilities of body weight and body length in females were significantly higher than those in males, indicating the females have higher selection potential. The high genetic correlations between females and males indicated that the growth of females and males are controlled by the same genes, such as *Mstn*.

## Methods

### Experimental materials

The experiment was conducted in the Mariculture Research Station of Yellow Sea Fisheries Research Institute, Chinese Academy of Fishery Sciences, located in Qingdao City, Shandong Province, China in 2018. The experimental shrimp were selected from 105 full-sib families (including 28 half sib families) of the 13^th^ generation (G_13_) of the selected population from our selective breeding program of *F. chinensis*. A nested mating design was used to produce the full- and half-sib families, and two females mated with one male to produce the half-sib families. The origin and the selection procedure of this selected population of *F. chinensis*, processes of family construction, hatching, and larvae rearing were described detailedly in our previous study^6^. The 105 families were reared separately in 200-L larvae-culture tanks after they were constructed. At the post-larvae stage in 35 days, 200 individuals of each family were moved to a larger tank (3 m^3^), and the families were still separately reared. When the average body weight reached about 2.0 g, each family was tagged with a unique family code by injecting visible implant fluorescent elastomers (VIE) at three locations on the 6^th^ and 5^th^ abdominal segment. After tagging for each family, 60 healthy tagged individuals were randomly selected and equally divided into four concrete tanks (100 m^2^) for rearing. During the rearing period, water was exchanged about 30% daily. The shrimp were fed with formulated diets four times every day, and a total daily supply accounted for 5% wet weight and adjusted daily. All the shrimp were individually tagged with numbered ring set on one ocular peduncle, and the family VIE tag, individual body weight (BW1) and sex were recorded at 190 days of culture (DOC).

In shrimp, the females grow faster than the males. In order to use enough females and males of each family for the next expression analysis, the families with lower survival rates (<50%) and with fewer females or males were eliminated. Finally, 44 full-sib families were selected, and about 20 individuals with a 1:1 sex ratio were selected from each family. All these selected individuals were moved to a cement tank (27 m^2^) with close recirculation system for communal rearing. During the rearing period, the temperature was 22–24 °C. In the same way, the shrimp were fed with formulated diets according to their wet weight and fresh *Ruditapes philippinarum*. The family VIE, individual tag, sex, body weight (BW2), and body length (BL2) were recorded at 230 DOC. At the same time, the muscle tissue of the healthy survival individuals was separately dissected and frozen immediately in liquid nitrogen, and after that they were stored at −80 °C until RNA extraction. Average daily gain (ADG) of body weight was calculated for each individual based on BW2 and BW1.

### RNA extraction and cDNA synthesis

RNA was extracted from muscle using the Trizol reagent (TAKARA, Japan) and phenol chloroform. The purity and concentration of the RNA was checked using a ND-1000 spectrophotometer (NANO DROP TECHNOLOGIES, USA), and the integrity of the RNA was monitored by electrophoresis on 1.5% agarose gel. Single-stand cDNA was synthesized from total RNA (1 μg) using PrimeScript RT reagent kit (TAKARA, Japan) following the manufacturer’s protocol. Then cDNA products were stored at −80 °C until the Real-time quantitative PCR (qRT-PCR).

### Expression analysis of *FcMstn*

The expression levels of the *F. chinensis Mstn* (*FcMstn*) in the muscle of the sampled individuals were analyzed by qRT-PCR. The specific primers (F: GATGCGACTGGCTTGAAACT, R: CGAATGAAGGAAGCTCCGAA, and Ta: 54 °C) of *FcMstn* for qRT-PCR were designed according to the open reading frame sequence of its complete cDNA (Accession number: MG437236, unreleased). The 18 S ribosomal RNA gene (F: TATACGCTAGTGGAGCTGGAA, R: GGGGAGGTAGTGACGAAAAAT, and Ta: 54 °C) of *F. chenensis* was used as the internal control^49^. Primers were tested to ensure specific amplification of single discrete band with no primer dimers. To detect optimal efficiency of the primer pair, the cDNA was analyzed with serial fold dilutions (1, 1/10, 1/20, 1/40 and 1/80). The qRT-PCR was performed in an ABI 7500 Sequence Detection System (ABI, USA) using a SYBR PrimeScript RT reagent Kit (TAKARA, Japan). Each reaction was performed in triplicate with the following conditions: 95 °C for 3 min, followed by 35 cycles of 95 °C for 30 s, 54 °C for 20 s, and 72 °C for 25 s. In order to ensure specificity of PCR product, melting curve determination was set at the end of each PCR reaction. The relative gene expression level of *FcMstn* for each individual was analyzed using the 2^−ΔΔCT^ method^50^.

### Variance components and heritability estimates

In order to modify the residual distribution, the *FcMstn* expression data were performed natural logarithm transformation. The descriptive statistics analysis for BW1, BW2, BL2, ADG, and *FcMstn* expression was carried out using the SPSS software (version 17.0). Means of BW1, BW2, BL2, ADG, and *FcMstn* expression between females and males were analyzed by independent sample *t*-test, respectively.

The analysis of variance indicated that sex showed significant effects on BW1, BW2, BL2, ADG, and *FcMstn* expression (*P* < 0.001). Age, pond and the initial body weight had no significant effect on BW1, BW2, BL2, ADG, and *FcMstn* expression (*P* > 0.05). In addition, body weight has a significant effect on *FcMstn* expression (*P* < 0.001), so BW1 as a covariate was included in the analysis model for *FcMstn* expression. The complete pedigree from G_0_ to G_12_ was used in the following analysis to account for the genetic relationships among the families and individuals.

The variance components and heritabilities for BW1, BW2, BL2, ADG, and *FcMstn* expression were estimated using ASREML 4.0^51^ at all-individual, female-individual, and male-individual levels, respectively. The fitted model for BW1, BW2, BL2, and ADG was Model 1, and that for *FcMstn* expression was Model 2, which were showed as follows:

Model 1 $${y}_{ij}=\mu +Se{x}_{i}+{a}_{j}+{e}_{ij}$$

Model 2 $${y}_{ij}=\mu +Se{x}_{i}+b\times BW{1}_{j}+{a}_{j}+{e}_{ij}$$

where *y*_*ij*_ is the observed value of BW1, BW2, BL2, ADG, or *FcMstn* expression of the *j*^th^ individual, $\mu$ is the overall mean, *Sex*_*i*_ is the fixed effect of the *i*^th^ gender, *BW*1_*j*_ is the covariate of the *j*^th^ body weight when the individual was sampled for gene expression analysis, *b* is the regression coefficient, *a*_*j*_ is the additive genetic effect of the *j*^th^ individual, and *e*_*ij*_ is the random residual error of the *j*^th^ individual, e ~ (0, I ${\sigma }_{e}^{2}$). The phenotypic variance (${\sigma }_{p}^{2}$) was taken as the sum of all of the variance components as follows: ${\sigma }_{p}^{2}={\sigma }_{a}^{2}+{\sigma }_{e}^{2}$. The heritability (*h*^2^) was calculated as the ratio between the genetic variance and the total phenotypic variance (${h}^{2}={\sigma }_{a}^{2}/{\sigma }_{p}^{2}$).

The *z-*scores were used to test whether the heritability estimates were significantly different from each other at all-individual, female-individual, and male-individual levels for each trait, and whether the heritability estimates were significantly different from zero^52^.

$$Z=\frac{{x}_{i}-{x}_{j}}{\sqrt{{\sigma }_{i}^{2}+{\sigma }_{j}^{2}}}$$

where ${x}_{i}$ and ${x}_{j}$ are the heritability estimates all-individual, female-individual, or male-individual levels, and ${\sigma }_{i}$ and ${\sigma }_{j}$ are their respective standard errors. Both ${x}_{j}$ and ${\sigma }_{j}$ were set to be zero when testing whether an estimate was significantly different from zero. The resulting *z*-score was then tested against a large-sample normal distribution.

### The phenotypic and genetic correlation estimates

The phenotypic (r_p_) and genetic (r_g_) correlations among BW1, BW2, BL2, ADG, and *FcMstn* expression, and r_g_ between females and males for the five traits were estimated using the bivariate animal model with the ASREML package^51^. The r_p_ and r_g_ were calculated for the above five traits at all-individual, female-individual, and male-individual levels, respectively. The statistical significances of r_g_ between the above traits at the three levels were estimated using the confidence intervals (CIs), which were calculated using 1.96 (i.e., [r_g_ − 1.96 × SE, r_g_ + 1.96 × SE]). If zero was contained within the CI, the difference between the r_g_ and zero was not significant. The statistical significances of r_p_ between the above traits at the three levels were also assessed using the confidence intervals (CIs).
